# HIV-1 p24 derived epitopes modulate KIR2DL2-binding to HLA-Cw03

**DOI:** 10.1186/1742-4690-9-S2-P180

**Published:** 2012-09-13

**Authors:** NH van Teijlingen, C Körner, L Fadda, C Brander, MN Carrington, D van Baarle, M Altfeld

**Affiliations:** 1 UMC Utrecht, Utrecht, the Netherlands; 2 Ragon Institute of MGH, MIT and Harvard, Boston, MA, USA; 3 IrsiCaixa (Institut de Recerca de la Sida), Barcelona, Spain; 4 National Cancer Institute at Frederick, Frederick, MD, USA

## Background

Recent studies have suggested that HIV-1 can evade Natural Killer (NK)-cell-mediated immunity by mutating viral epitopes to enhance engagement of inhibitory Killer Ig-like receptors (KIRs) expressed on NK cells. However, the precise mechanisms modulating the interaction of inhibitory KIRs and their HLA class I ligands, and the role that HIV-1 epitopes might play in this interaction are not well understood. In this study we investigated whether HLA-Cw3-presented epitopes within HIV-1 p24 Gag can modulate binding of KIR2DL2, an inhibitory KIR, to HLA-Cw03.

## Methods

Using tapasin-deficient 721.220 cell line expressing HLA-Cw*0304 we initially screened for HIV-1 peptides that stabilized HLA-Cw*0304 expression using 222 10-mer peptides overlapping by 9 amino acids spanning the entire HIV-1 p24 Gag sequence. Peptides stabalizing HLA-Cw*0304 expression were thereafter investigated for their ability to facilitate binding of a KIR2DL2-IgG fusion construct.

## Results

We identified several HIV-1 p24 epitopes that were able to stabilize HLA–Cw*0304 expression. A subset of these epitopes also allowed for binding of KIR2DL2. Currently we are investigating the consequences of KIR2DL2-binding to HLA class I presented HIV-1 epitopes for the antiviral function of primary NK cells from KIR2DL2+ individuals.

## Conclusion

Taken together, these studies have identified epitopes within HIV-1 that enhance the binding of the inhibitory NK cell receptor KIR2DL2 to its ligand HLA-Cw3, and are in line with recent data suggesting that the sequence of the HLA class I presented epitope has an important impact on the interaction between KIR and HLA class I (Boyington et al. Nature 2000, Vivian et al. Nature 2011).

